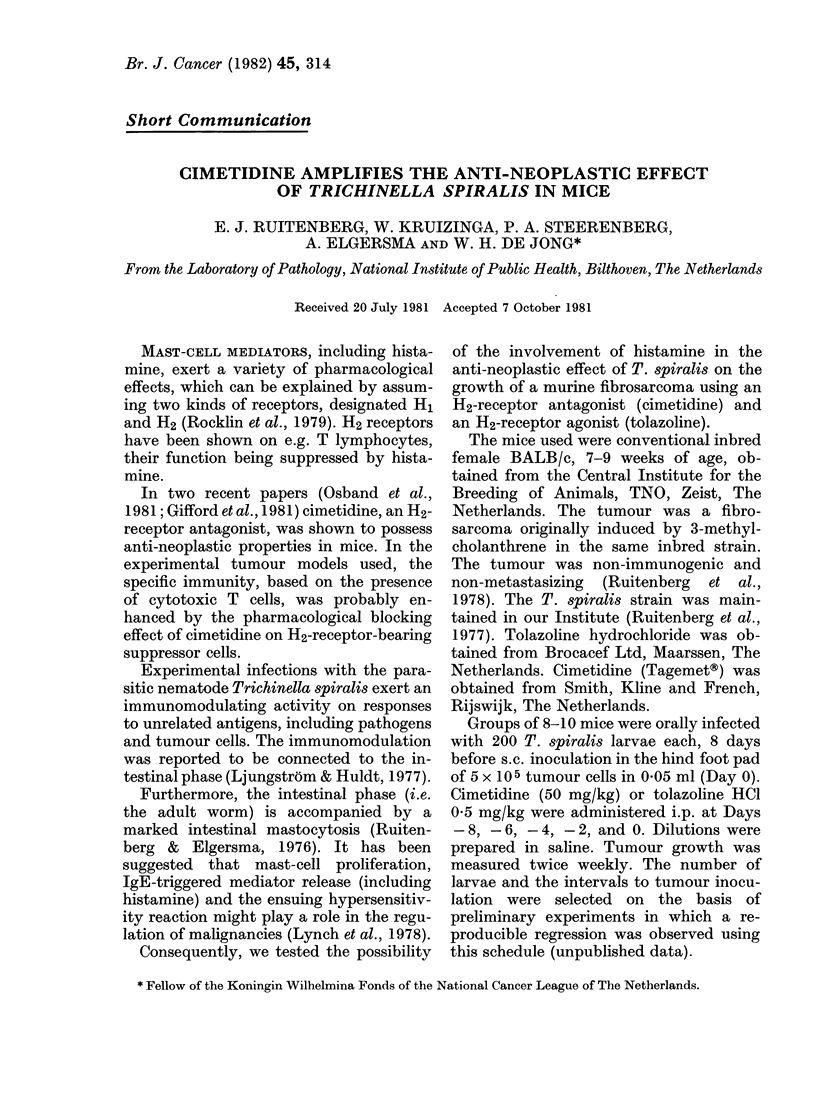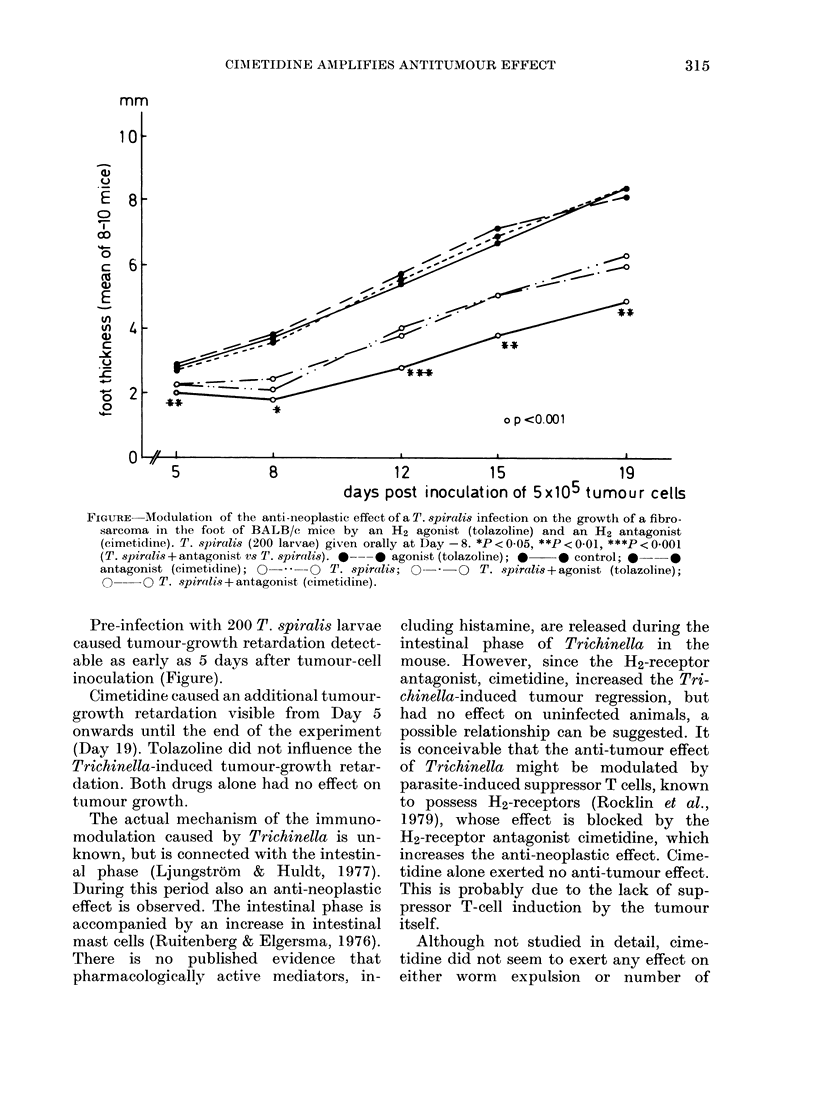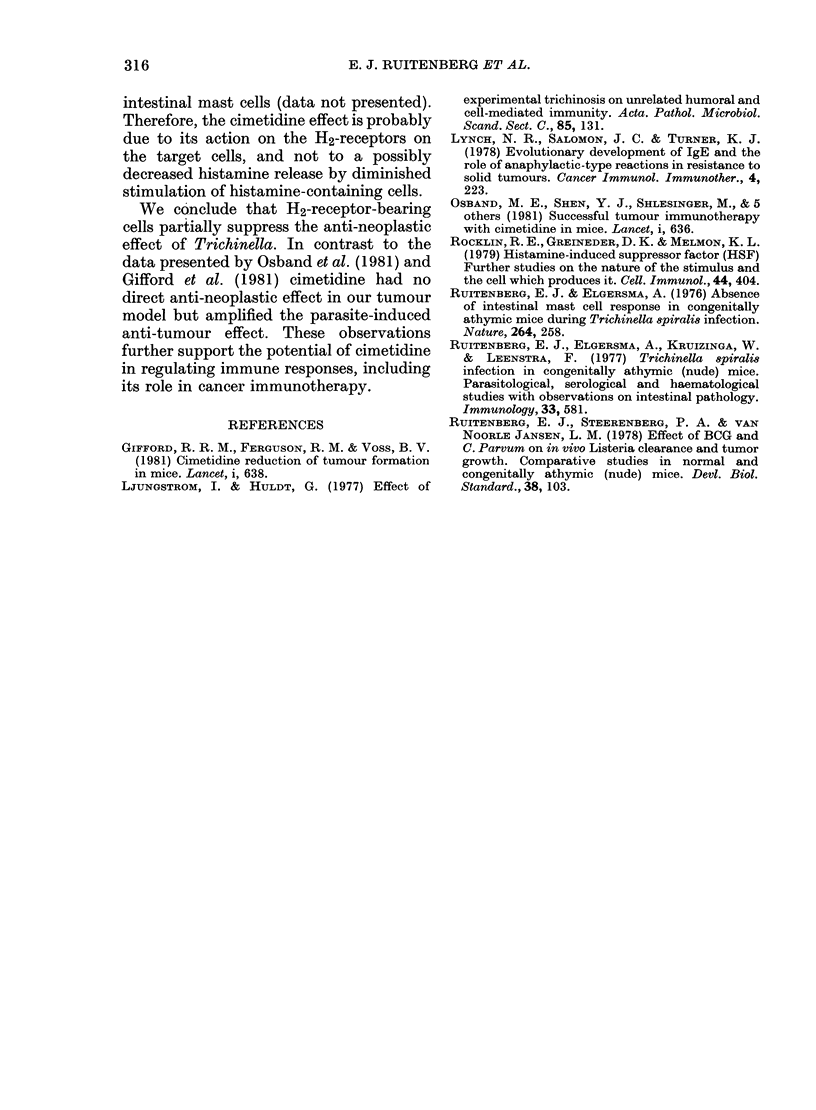# Cimetidine amplifies the anti-neoplastic effect of Trichinella spiralis in mice.

**DOI:** 10.1038/bjc.1982.50

**Published:** 1982-02

**Authors:** E. J. Ruitenberg, W. Kruizinga, P. A. Steerenberg, A. Elgersma, W. H. De Jong


*Br. J. Cancer* (1982) **45**, 314

***Short Communication***

### CIMETIDINE AMPLIFIES THE ANTI-NEOPLASTIC EFFECT OF *TRICHINELLA SPIRALIS* IN MICE

E. J. RUITENBERG, W. KRUIZINGA, P. A. STEERENBERG, A. ELGERSMA AND W. H. DE JONG*

*From the Laboratory of Pathology, National Institute of Public Health, Bilthoven, The Netherlands*

Received 20 July 1981 Accepted 7 October 1981

MAST-CELL MEDIATORS, including histamine, exert a variety of pharmacological effects, which can be explained by assuming two kinds of receptors, designated $H_1$ and $H_2$ (Rocklin *et al.*, 1979). $H_2$ receptors have been shown on e.g. T lymphocytes, their function being suppressed by histamine.

In two recent papers (Osband *et al.*, 1981; Gifford *et al.*, 1981) cimetidine, an $H_2$-receptor antagonist, was shown to possess anti-neoplastic properties in mice. In the experimental tumour models used, the specific immunity, based on the presence of cytotoxic T cells, was probably enhanced by the pharmacological blocking effect of cimetidine on $H_2$-receptor-bearing suppressor cells.

Experimental infections with the parasitic nematode *Trichinella spiralis* exert an immunomodulating activity on responses to unrelated antigens, including pathogens and tumour cells. The immunomodulation was reported to be connected to the intestinal phase (Ljungström & Huldt, 1977).

Furthermore, the intestinal phase (*i.e.* the adult worm) is accompanied by a marked intestinal mastocytosis (Ruitenberg & Elgersma, 1976). It has been suggested that mast-cell proliferation, IgE-triggered mediator release (including histamine) and the ensuing hypersensitivity reaction might play a role in the regulation of malignancies (Lynch *et al.*, 1978).

Consequently, we tested the possibility of the involvement of histamine in the anti-neoplastic effect of *T. spiralis* on the growth of a murine fibrosarcoma using an $H_2$-receptor antagonist (cimetidine) and an $H_2$-receptor agonist (tolazoline).

The mice used were conventional inbred female BALB/c, 7–9 weeks of age, obtained from the Central Institute for the Breeding of Animals, TNO, Zeist, The Netherlands. The tumour was a fibrosarcoma originally induced by 3-methylcholanthrene in the same inbred strain. The tumour was non-immunogenic and non-metastasizing (Ruitenberg *et al.*, 1978). The *T. spiralis* strain was maintained in our Institute (Ruitenberg *et al.*, 1977). Tolazoline hydrochloride was obtained from Brocacef Ltd, Maarssen, The Netherlands. Cimetidine (Tagemet®) was obtained from Smith, Kline and French, Rijswijk, The Netherlands.

Groups of 8–10 mice were orally infected with 200 *T. spiralis* larvae each, 8 days before s.c. inoculation in the hind foot pad of $5 \times 10^5$ tumour cells in 0·05 ml (Day 0). Cimetidine (50 mg/kg) or tolazoline HCl 0·5 mg/kg were administered i.p. at Days $-8$, $-6$, $-4$, $-2$, and 0. Dilutions were prepared in saline. Tumour growth was measured twice weekly. The number of larvae and the intervals to tumour inoculation were selected on the basis of preliminary experiments in which a reproducible regression was observed using this schedule (unpublished data).

* Fellow of the Koningin Wilhelmina Fonds of the National Cancer League of The Netherlands.

FIGURE—Modulation of the anti-neoplastic effect of a *T. spiralis* infection on the growth of a fibrosarcoma in the foot of BALB/c mice by an $H_2$ agonist (tolazoline) and an $H_2$ antagonist (cimetidine). *T. spiralis* (200 larvae) given orally at Day −8. $^*P<0{\cdot}05$, $^{**}P<0{\cdot}01$, $^{***}P<0{\cdot}001$ (*T. spiralis*+antagonist *vs T. spiralis*). ●– – –● agonist (tolazoline); ●——● control; ●——● antagonist (cimetidine); ○—··—○ *T. spiralis*; ○—·—○ *T. spiralis*+agonist (tolazoline); ○——○ *T. spiralis*+antagonist (cimetidine).

Pre-infection with 200 *T. spiralis* larvae caused tumour-growth retardation detectable as early as 5 days after tumour-cell inoculation (Figure).

Cimetidine caused an additional tumour-growth retardation visible from Day 5 onwards until the end of the experiment (Day 19). Tolazoline did not influence the *Trichinella*-induced tumour-growth retardation. Both drugs alone had no effect on tumour growth.

The actual mechanism of the immunomodulation caused by *Trichinella* is unknown, but is connected with the intestinal phase (Ljungström & Huldt, 1977). During this period also an anti-neoplastic effect is observed. The intestinal phase is accompanied by an increase in intestinal mast cells (Ruitenberg & Elgersma, 1976). There is no published evidence that pharmacologically active mediators, including histamine, are released during the intestinal phase of *Trichinella* in the mouse. However, since the $H_2$-receptor antagonist, cimetidine, increased the *Trichinella*-induced tumour regression, but had no effect on uninfected animals, a possible relationship can be suggested. It is conceivable that the anti-tumour effect of *Trichinella* might be modulated by parasite-induced suppressor T cells, known to possess $H_2$-receptors (Rocklin *et al.*, 1979), whose effect is blocked by the $H_2$-receptor antagonist cimetidine, which increases the anti-neoplastic effect. Cimetidine alone exerted no anti-tumour effect. This is probably due to the lack of suppressor T-cell induction by the tumour itself.

Although not studied in detail, cimetidine did not seem to exert any effect on either worm expulsion or number of intestinal mast cells (data not presented). Therefore, the cimetidine effect is probably due to its action on the $H_2$-receptors on the target cells, and not to a possibly decreased histamine release by diminished stimulation of histamine-containing cells.

We conclude that $H_2$-receptor-bearing cells partially suppress the anti-neoplastic effect of *Trichinella*. In contrast to the data presented by Osband *et al.* (1981) and Gifford *et al.* (1981) cimetidine had no direct anti-neoplastic effect in our tumour model but amplified the parasite-induced anti-tumour effect. These observations further support the potential of cimetidine in regulating immune responses, including its role in cancer immunotherapy.

### REFERENCES

GIFFORD, R. R. M., FERGUSON, R. M. & VOSS, B. V. (1981) Cimetidine reduction of tumour formation in mice. *Lancet*, i, 638.

LJUNGSTROM, I. & HULDT, G. (1977) Effect of experimental trichinosis on unrelated humoral and cell-mediated immunity. *Acta. Pathol. Microbiol. Scand. Sect. C.*, **85**, 131.

LYNCH, N. R., SALOMON, J. C. & TURNER, K. J. (1978) Evolutionary development of IgE and the role of anaphylactic-type reactions in resistance to solid tumours. *Cancer Immunol. Immunother.*, **4**, 223.

OSBAND, M. E., SHEN, Y. J., SHLESINGER, M., & 5 others (1981) Successful tumour immunotherapy with cimetidine in mice. *Lancet*, i, 636.

ROCKLIN, R. E., GREINEDER, D. K. & MELMON, K. L. (1979) Histamine-induced suppressor factor (HSF) Further studies on the nature of the stimulus and the cell which produces it. *Cell. Immunol.*, **44**, 404.

RUITENBERG, E. J. & ELGERSMA, A. (1976) Absence of intestinal mast cell response in congenitally athymic mice during *Trichinella spiralis* infection. *Nature*, **264**, 258.

RUITENBERG, E. J., ELGERSMA, A., KRUIZINGA, W. & LEENSTRA, F. (1977) *Trichinella spiralis* infection in congenitally athymic (nude) mice. Parasitological, serological and haematological studies with observations on intestinal pathology. *Immunology*, **33**, 581.

RUITENBERG, E. J., STEERENBERG, P. A. & VAN NOORLE JANSEN, L. M. (1978) Effect of BCG and *C. Parvum* on *in vivo* Listeria clearance and tumor growth. Comparative studies in normal and congenitally athymic (nude) mice. *Devl. Biol. Standard.*, **38**, 103.